# Insulin regulates Rab3–Noc2 complex dissociation to promote GLUT4 translocation in rat adipocytes

**DOI:** 10.1007/s00125-015-3627-3

**Published:** 2015-05-30

**Authors:** Francoise Koumanov, Vinit J. Pereira, Judith D. Richardson, Samantha L. Sargent, Daniel J. Fazakerley, Geoffrey D. Holman

**Affiliations:** Department of Biology and Biochemistry, University of Bath, Bath, BA2 7AY UK; Charles Perkins Centre, School of Molecular Bioscience, The University of Sydney, Sydney, NSW Australia

**Keywords:** GLUT4, G-protein photolabel, Insulin, Noc2, Rab3, *Rph3al*

## Abstract

**Aims/hypothesis:**

The glucose transporter GLUT4 is present mainly in insulin-responsive tissues of fat, heart and skeletal muscle and is translocated from intracellular membrane compartments to the plasma membrane (PM) upon insulin stimulation. The transit of GLUT4 to the PM is known to be dependent on a series of Rab proteins. However, the extent to which the activity of these Rabs is regulated by the action of insulin action is still unknown. We sought to identify insulin-activated Rab proteins and Rab effectors that facilitate GLUT4 translocation.

**Methods:**

We developed a new photoaffinity reagent (Bio-ATB-GTP) that allows GTP-binding proteomes to be explored. Using this approach we screened for insulin-responsive GTP loading of Rabs in primary rat adipocytes.

**Results:**

We identified Rab3B as a new candidate insulin-stimulated G-protein in adipocytes. Using constitutively active and dominant negative mutants and Rab3 knockdown we provide evidence that Rab3 isoforms are key regulators of GLUT4 translocation in adipocytes. Insulin-stimulated Rab3 GTP binding is associated with disruption of the interaction between Rab3 and its negative effector Noc2. Disruption of the Rab3–Noc2 complex leads to displacement of Noc2 from the PM. This relieves the inhibitory effect of Noc2, facilitating GLUT4 translocation.

**Conclusions/interpretation:**

The discovery of the involvement of Rab3 and Noc2 in an insulin-regulated step in GLUT4 translocation suggests that the control of this translocation process is unexpectedly similar to regulated secretion and particularly pancreatic insulin-vesicle release.

**Electronic supplementary material:**

The online version of this article (doi:10.1007/s00125-015-3627-3) contains peer-reviewed but unedited supplementary material, which is available to authorised users.

## Introduction

Rab proteins switch between active and inactive states and thereby control the vesicular traffic through multiple, inter-related membrane compartments [1, 2]. Studies using fluorescence-domain-tagged Rabs have enabled these proteins to be traced in distinct subcellular compartments [2–4]. Rab localisations are dependent on the coordinated control by Rab guanosine exchange factors (Rab-GEFs), which are responsible for loading with GTP, and Rab GTPase activating proteins (Rab-GAPs), which increase the unloading through activating the GTPase activity of the Rabs [3, 5]. Consequently, Rab proteins have distinct subcellular localisations that are associated with specific functions. For example, a screen of Rabs 1–41 in PC12 cells revealed that only Rab3 and Rab27 were key components of the exocytosis of hormone-containing secretory granules in PC12 cells [6]. Understanding the GTP loading state of Rabs is particularly important in studies of membrane traffic pathways that are regulated hormonally, such as insulin-regulated GLUT4 traffic [4, 7, 8].

**Fig. 1:**
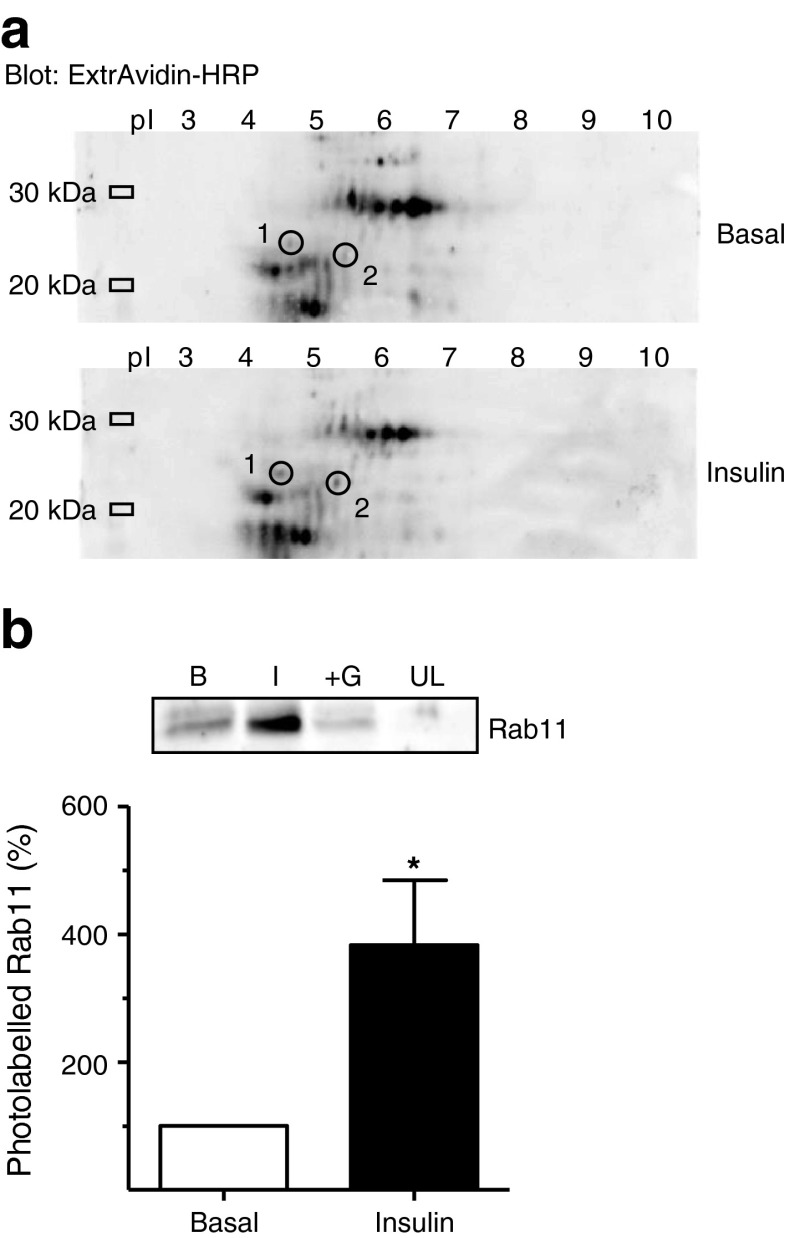
Identification of Rab3B as an insulin-sensitive GTPase in rat adipocytes. (**a**) Two-dimensional gel analysis of Bio-ATB-GTP-labelled total membranes. Biotinylated proteins were blotted with ExtrAvidin–HRP (Sigma, St. Louis, MO, USA). Circled spots 1 (Rab3B) and 2 (Rab11) indicate proteins for which the biotin signal was identified as increasing upon insulin stimulation. (**b**) Bio-ATB-GTP photolabelling of Rab11 in total-membrane preparations from rat adipocytes. Biotinylated proteins were precipitated with streptavidin and immunoblotted with antibodies against Rab11. Data are means ± SEM from at least three independent experiments. *p < 0.05 vs basal. B, basal; I, insulin; +G, excess GTP; UL, unlabelled (without Bio-ATB-GTP)

The intracellular sequestration of the insulin-sensitive glucose transporter isoform GLUT4 is required for the generation of a storage compartment from which it can be recruited to the plasma membrane (PM) rapidly upon activation of insulin signalling [9]. The insulin-dependent movement of GLUT4 from its reservoir compartment is dependent on several G-proteins, which may be activated by insulin signalling. To explore insulin-dependent G-protein activation we have developed a new reagent Bio-ATB-GTP with a biotin tag, a photoreactive azitrifluoroethylbenzoyl (ATB) group and a GTP moiety (see electronic supplementary material [ESM] Fig. 1a). Several approaches using Bio-ATB-GTP are available to detect activation of the GTP loading state potential of Rabs. We describe an unbiased and generic approach that utilises Bio-ATB-GTP in tandem with mass spectrometry to identify G-proteins that are GTP loaded in response to insulin. Using this approach we have identified that Rab3B is activated by insulin in rat adipocytes and acts in conjunction with Noc2 to modulate GLUT4 translocation.

## Methods

A detailed description of all experimental procedures, antibodies, cDNA constructs and image analysis is provided in the ESM Methods.

### Quantification of ***Rab3*** isoform and ***Noc2*** mRNA levels

Total RNA was extracted from rat tissues with TriPure reagent (Roche Diagnostics, Mannheim, Germany) and from 3T3-L1 adipocytes using the SV Total RNA isolation system (Promega, Madison, WI, USA). Five-hundred nanogrammes of total RNA, treated with DNaseI, was reverse transcribed to cDNA and 5 ng cDNA and 500 nmol/l primers (ESM Table 1) were used in the quantitative RT-PCR reaction performed in a StepOnePlus Real-Time PCR System (Applied Biosystems, Life Technologies, Paisley, UK) using iTaq SYBR Green supermix with ROX (BioRad, Hercules, CA, USA). The comparative C_t_ method was used to quantify the relative expression of *Rab3* in rat tissues and 3T3-L1 adipocytes.

### Rat adipose cell isolation and fractionation

Adipose cells from epididymal fat pads of male Wistar rats (colony bred at the University of Bath, fed on a standard chow diet), weighing 180–200 g, were prepared by collagenase digestion as described previously [10]. Cells were maintained at 37°C in Krebs–Ringer-HEPES (KRH) buffer supplemented with 1% (*w*/*v*) BSA. Adipocytes were electroporated with pCis2 hemagglutinin (HA)-*Glut4* [11] alone or together with pcDNA3.1-*Rab3B* wild-type (WT), Q81L or T36N or p3xFLAG-*Rab3B* WT, Q81L or T36N or p3xFLAG-*Noc2* WT or *Noc2*^W154A, F155A, Y156A^ (Noc2^AAA^); (*Glut4* is also known as *Slc2a4*; *Noc2* is also known as *Rph3al*). Cell-surface HA-GLUT4 was detected following the method described [11, 12].

Preparations of an adipocyte total cell membrane pellet and membrane subfractionation were performed according to the method described by Simpson et al [13]. GLUT4-containing vesicles were isolated following the protocol described previously [14].

A whole-cell lysate was obtained by washing the cells twice in KRH buffer and then lysing them for 20 min at 18°C in lysis buffer. Lysates were cleared by centrifugation at 17,000*g* at 4°C.

### Bio-ATB-GTP photolabel synthesis and labelling

The Bio-ATB-GTP photolabel was synthesised following a procedure described in detail in the ESM Methods. The product was obtained in a yield of approximately 30%. The Bio-ATB-GTP structure (ESM Fig. 1) was confirmed by mass spectroscopy (Bruker, Coventry, UK, MicroTOF, +ve ion). Predicted for C44H63F3N13O22P3S: 1307.3096; Found: 1308.3169 (MH+). Bio-ATB-GTP (40 μmol/l) was added to the membranes (300 μg) and incubated for 30 min on ice with occasional mixing in the presence of EDTA. MgCl_2_ was added and membranes were irradiated twice for 1 min in a UV chamber at a wavelength of 350 nm. The biotinylated photolabelled membranes were washed, solubilised and then precipitated overnight with streptavidin agarose at 4°C. Biotinylated proteins were separated by SDS-PAGE and immunoblotted with the relevant Rab antibodies or were resolved by two-dimensional gel electrophoresis. Biotinylated proteins of interested were detected by avidin blotting and analysed by trypsin digestion and mass spectroscopy.

### Effects of siRNA silencing in 3T3-L1 cells

3T3-L1 fibroblasts were cultured in DMEM and differentiated to adipocytes by treatment with insulin, dexamethasone and isobutylmethylxanthine. 3T3-L1 adipocytes at day 3 of differentiation were used for small interfering RNA (siRNA) transfection as described [15]. Uptake of 50 μmol/l 2-deoxy[^3^*H*]glucose was measured for 5 min with and without 100 nmol/l insulin. Bio-azitrifluoroethylbenzoyl-bis-glucose-propyl-2-amine (Bio-ATB-BGPA) photolabelling (300 μmol/l final concentration) was determined by irradiation for 1 min in a UV chamber at a wavelength of 300 nm [16]. Biotinylated transporters were solubilised, precipitated with streptavidin agarose, separated by SDS-PAGE and then detected with either GLUT1- or GLUT4-specific antibodies.

### Rab3 and Noc2 pull-down experiments

FLAG-*Rab3B*^Q81L^ or FLAG-*Rab3B*^T36N^ were expressed in HEK293T cells and immobilised with anti-FLAG antibody-conjugated agarose beads. After extensive washes the beads were incubated for 2 h with whole-cell adipocyte lysate prepared from basal or insulin-stimulated cells. Rab3B-interacting proteins were eluted with high-salt elution buffer. Eluted samples were then desalted, resolved by SDS-PAGE and analysed by immunoblotting. GST-Noc2 WT or GST-Noc2^AAA^, or maltose-binding protein (MBP)-Noc2 WT, were immobilised on glutathione or amylose beads, respectively. Basal or insulin-treated lysate from adipocytes overexpressing FLAG-Rab3B were incubated with the Noc2-bound beads for 2 h at 4°C. FLAG-Rab3B that bound to the beads was analysed by SDS-PAGE and immunoblotting.

### Statistical analysis

Results were analysed using two-tailed un-paired *t* tests. A *p* value <0.05 was considered statistically different.

## Results

### Characterisation of insulin-regulated small GTPases using Bio-ATB-GTP

We explored the use of Bio-ATB-GTP in combination with proteomic analysis to identify small G-proteins that differ in their GTP-loadable state in an insulin-dependent manner. Bio-ATB-GTP-photolabelled adipocyte membranes were separated by two-dimensional gel electrophoresis (Fig. 1a). Biotinylated-protein spots where the intensity of the signal varied between the basal and insulin-stimulated states were then aligned with the protein that remained in the stained gel. Matrix-assisted laser desorption/ionisation–time of flight (MALDI-TOF) MS identified Rab3B as an insulin-sensitive GTPase (ESM Table 2). Tryptic peptides were additionally analysed by nano-LC MS/MS. Rab3B was identified (spot1 on Fig. 1a and ESM Table 1) together with Rab GTPase Rab11B (spot2 on Fig. 1a and ESM Table 2). Densitometric analysis of the intensity of the signals detected on the two-dimensional gels for both Rab3B and Rab11B revealed an enrichment of two- to threefold following insulin stimulation. The latter increase corresponded with the level of stimulation observed when using a specific Rab11 antibody in an antibody-targeted application of this photolabelling approach (Fig. 1b and [17]). Bio-ATB-GTP has been used to tag a range of G-proteins (including the non-Rab proteins TC10 [18] and RalA [19], ESM Fig. 2), which show varying degrees of insulin activation.

### Insulin stimulation of the GTP loading state potential of Rab3B in rat adipocytes

**Fig. 2:**
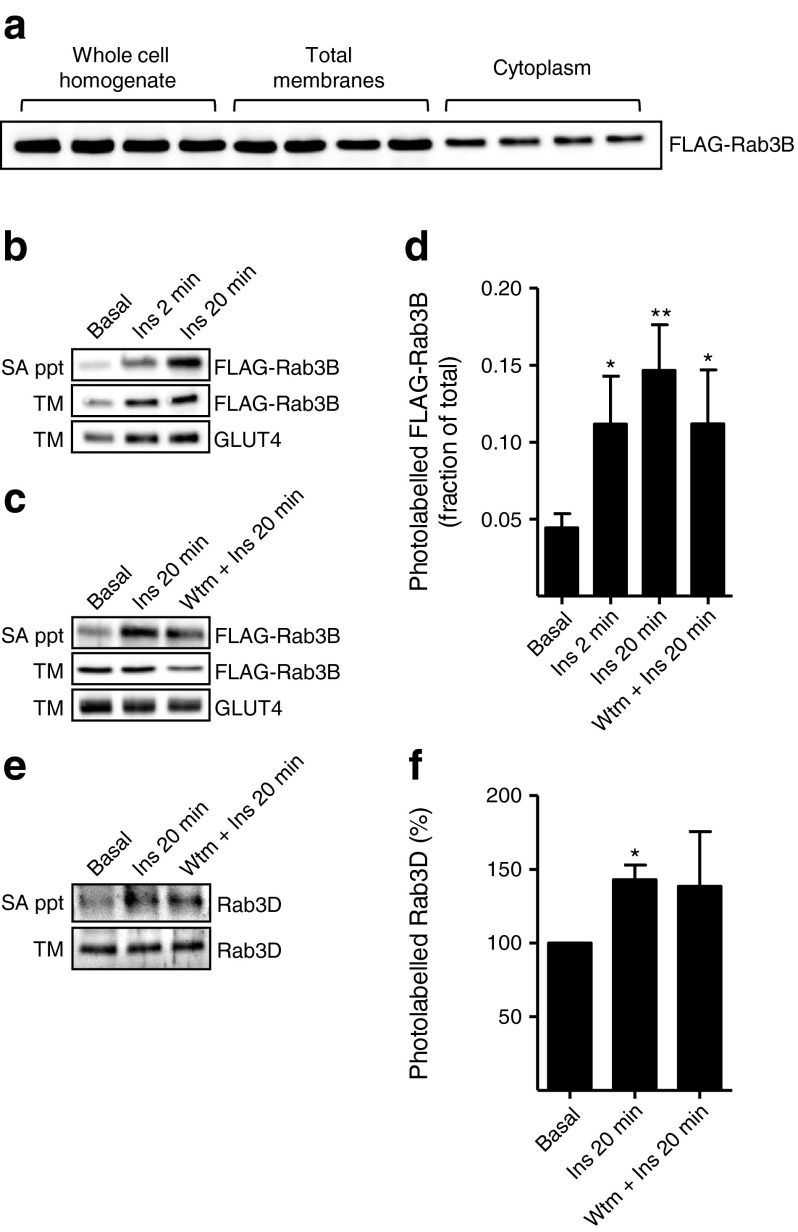
FLAG-Rab3B and endogenous Rab3D are activated upon insulin stimulation. (**a**) Distribution of expressed FLAG-Rab3B between the total-membrane and the cytoplasmic fractions of rat adipocytes. (**b**) Insulin stimulation (Ins) of the Bio-ATB-GTP loading state of FLAG-Rab3B. Streptavidin-precipitated proteins were detected with anti-FLAG antibody. (**c**) Effect of wortmannin (200 nmol/l for 10 min) (Wtm) on insulin-stimulated FLAG-Rab3B GTP loading. (**d**) Quantification of the data presented in (**b**) and (**c**). Data are means ± SEM from three independent experiments. *p < 0.05 vs basal. (**e**) Effect of insulin stimulation on endogenous Rab3D GTP loading. Total-membrane preparations (300 μg/condition) were labelled with Bio-ATB-GTP and streptavidin-precipitated proteins immunoblotted with anti-Rab3D antibody. (**f**) Quantification of the data shown in (**e**). Data are means ± SEM from three independent experiments. *p < 0.05 vs basal. SA ppt, streptavidin precipitation; TM, total-membrane loading control

As no suitable commercially available antibodies to rodent Rab3B were available, FLAG-tagged rat Rab3B was expressed in rat adipocytes and was found to be strongly associated with membrane fractions (approximately 70%, Fig. 2a). We used Bio-ATB-GTP to tag membrane preparations isolated from basal or insulin-stimulated rat adipocytes expressing FLAG-Rab3B. A time-dependent activation of Rab3B loading was observed following treatment with insulin (Fig. 2b, d). Activation of Rab3B was independent of phosphoinositide 3 (PI 3)-kinase activity as wortmannin failed to inhibit Rab3B loading (Fig. 2c, d). Rab3D was previously detected in 3T3-L1 adipocytes [20] and has been shown to be present in insulin-responsive tissues [21]. We therefore explored whether this isoform also exhibited insulin-dependent GTP loading. We observed an increase in the GTP loading state of Rab3D (Fig. 2e, f). Wortmannin treatment of the cells before membrane isolation did not inhibit insulin-dependent stimulation of GTP loading of Rab3D. These data are consistent with PI 3-kinase-independent activation of both Rab3B and Rab3D.

### Rab3B involvement in GLUT4 translocation in rat adipocytes

**Fig. 3:**
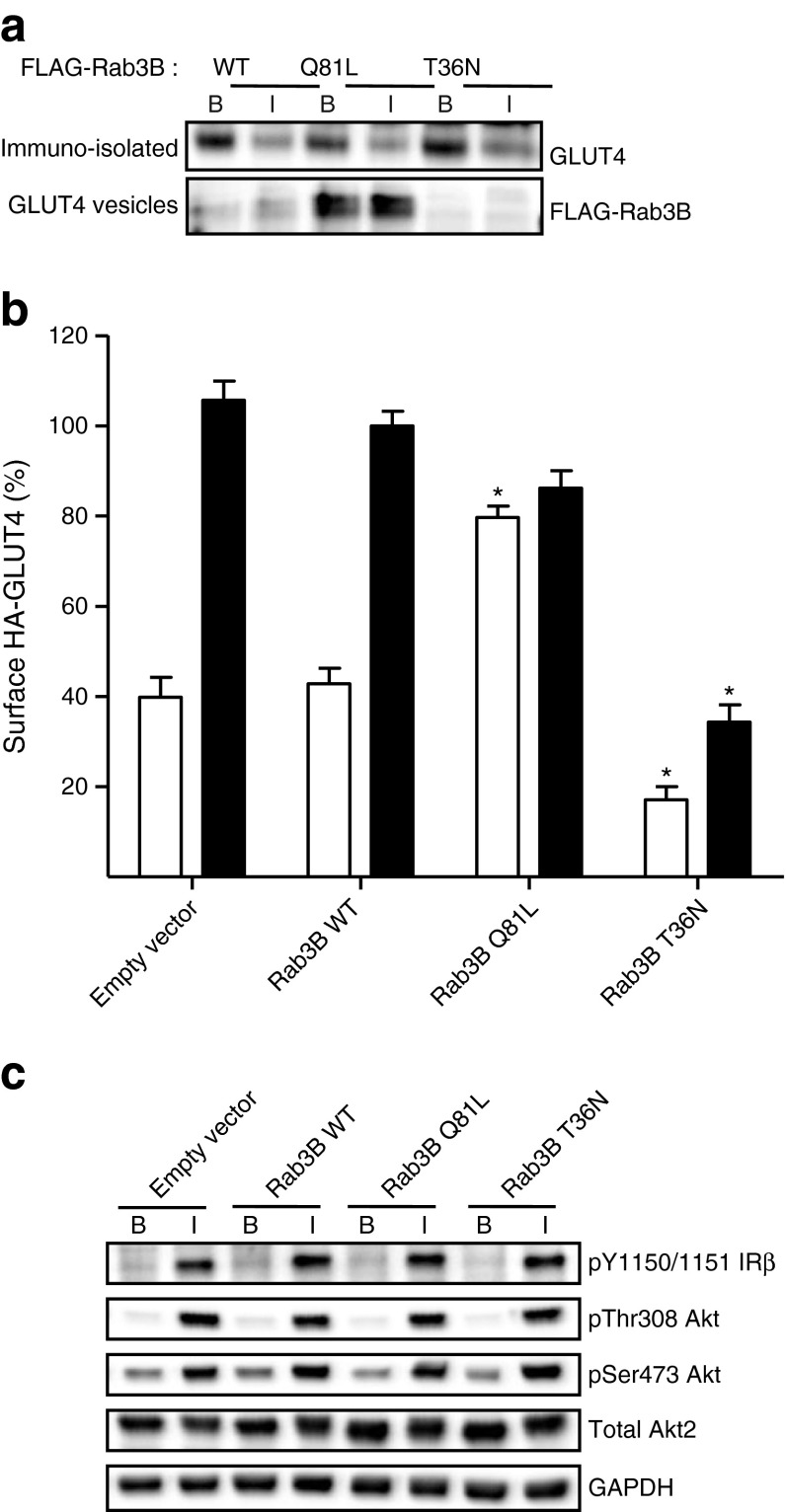
Rab3B modulates GLUT4 vesicle translocation. (**a**) Isolated GLUT4 vesicles from rat adipocytes expressing FLAG-*Rab3B*-WT, FLAG-*Rab3B*-Q81L or FLAG-*Rab3B*-T36N cDNA were separated by SDS-PAGE, and immunoblotted with anti-GLUT4 or with anti-FLAG antibodies. A representative immunoblot from three independent experiments is shown. B, basal; I, insulin. (**b**) Effect of constitutively active *Rab3B*-Q81L on HA-GLUT4 present at the cell surface. Rat adipocytes were co-transfected with HA-*Glut4* cDNA in combination with pcDNA3.1 empty vector, *Rab3B*-WT, *Rab3B*-Q81L or *Rab3B*-T36N cDNA. Data are means ± SEM from four independent experiments. *p < 0.05 vs empty vector control. White bars, basal; black bars, insulin. (**c**) Insulin signalling in rat adipocytes electroporated with Rab3B constructs. Representative immunoblots from two experiments are shown of phosphorylated-state specific antibodies against insulin receptor β subunit (pY1150/1151 IRβ) and Akt (pThr308 Akt and pSer473 Akt) in basal (B) and insulin-stimulated (I) cells

Only low levels of FLAG-Rab3B associated with the GLUT4 vesicles (Fig. 3a). The association with GLUT4 storage vesicles (GSVs) was slightly higher in the insulin-stimulated state. Expression of the constitutively active mutant of Rab3B (FLAG-*Rab3B*^Q81L^) in adipocytes resulted in a marked association of this mutant protein with GLUT4 vesicles.

We next investigated whether overexpression of Rab3B constructs affected insulin-stimulated translocation of GLUT4 to the cell surface. WT, Q81L and T36N mutants of Rab3B were expressed in rat adipocytes together with exofacial HA-epitope tagged GLUT4. Expression of the WT Rab3B did not affect the level of HA-GLUT4 that was exposed at the surface of either basal or insulin-stimulated adipocytes (Fig. 3b). However, expression of Rab3B^Q81L^ markedly increased the level of HA-GLUT4 detected at the cell surface of unstimulated cells to a level equal to that occurring upon insulin stimulation of the cells. Expression of the GDP-binding mutant Rab3B^T36N^ decreased the levels of HA-GLUT4 present at the cell surface of adipocytes both in the basal and insulin-stimulated state. Expression of the recombinant Rab3B in adipocytes did not affect early insulin signalling (Fig. 3c).

### Rab3 isoform silencing and GLUT4 translocation

Analysis of the mRNA levels of the four Rab3 isoforms in insulin-sensitive tissues by quantitative RT-PCR revealed that *Rab3B* and *Rab3D* are the most abundant isoforms in rat adipocytes (ESM Fig. 3). In contrast *Rab3A* and *Rab3D* were the most abundant isoforms in mouse 3T3-L1 adipocytes, suggesting interspecies differences or a difference between primary adipose cells and this cell line. RNA silencing is difficult to achieve in primary rat adipocytes and this difficulty is compounded by the presence of multiple Rab3 isoforms with likely functional redundancy. Therefore, to investigate whether silencing Rab3 affected insulin-regulated GLUT4 translocation, we examined Rab3 isoform silencing in 3T3-L1 adipocytes.

**Fig. 4:**
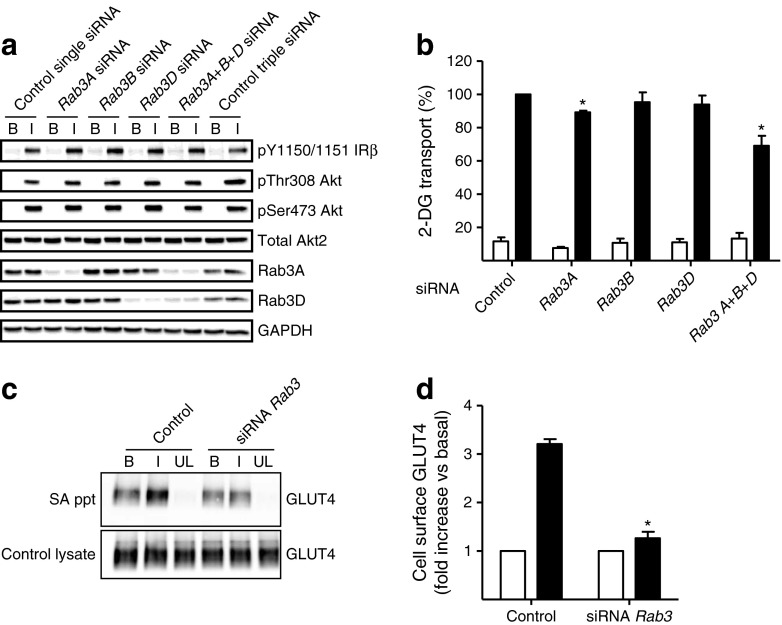
Rab3 silencing in 3T3-L1 adipocytes decreases glucose uptake and GLUT4 translocation. (**a**) Immunoblot of *Rab3A* and *Rab3D* siRNA silencing and insulin-signalling components (as described, Fig. 3c) in 3T3-L1 adipocytes. 3T3-L1 adipocytes (day 3 of differentiation) were transfected with siRNA against *Rab3A*, *Rab3B* and *Rab3D* (separate or combined). Representative immunoblots from three experiments are shown. Control cells were transfected with equal amounts of non-targeting siRNA. (**b**) Effect of siRNA silencing of Rab3 isoforms on insulin-stimulated 2-deoxy-d-glucose (2-DG) uptake. Data are means ± SEM from three independent experiments. *p < 0.05 vs non-targeting siRNA. White bars, basal; black bars, insulin. (**c**) Effect of siRNA silencing of Rab3 isoforms on GLUT4 translocation to the cell surface. 3T3-L1 adipocytes (basal or 100 nmol/l insulin for 30 min) were labelled with Bio-ATB-BGPA. Solubilised and streptavidin-precipitated proteins were immunoblotted for GLUT4. SA ppt, streptavidin precipitation, B, basal; I, insulin, UL, no photolabel. (**d**) Quantification of the data shown in (**c**). Data are means ± SEM from three independent experiments. *p < 0.05 vs control. White bars, basal; black bars, insulin

Rab3A and Rab3D protein levels both increased in 3T3-L1 adipocytes over 10 days of differentiation and this increase was associated with similarly increased expression of GLUT4 over the same time course [22]. siRNA-mediated knockdown of *Rab3A* and *Rab3D* resulted in a 60%–70% reduction of Rab3A and Rab3D protein levels and this occurred without an effect on early insulin signalling to the insulin receptor or to PI 3-kinase (Fig. 4a). We monitored the uptake of 2-deoxy-d-glucose into cells silenced for the Rab3 isoforms as indicated (Fig. 4b). siRNA silencing of the individual Rab3 isoforms did not produce large inhibitory effects on 2-deoxy-d-glucose uptake, although Rab3A silencing marginally but significantly decreased insulin-stimulated 2-deoxy-d-glucose uptake. When all three isoforms (Rab3A, B and D) were simultaneously knocked down, a 35% decrease in insulin-stimulated 2-deoxy-d-glucose uptake was observed. Therefore, loss of individual isoforms may be functionally compensated for by other isoforms.

To assess more directly whether GLUT4 translocation was affected by the RNA silencing of Rab3, we used the glucose transporter affinity tag Bio-ATB-BGPA to label the cell-surface exposed GLUT4. These experiments indicated that insulin-stimulated GLUT4 translocation was inhibited by over 80% when all three Rab3 isoforms were silenced (Fig. 4c, d). The triple isoform knockdown of Rab3 did not affect the level of translocation of GLUT1 to the cell surface of the 3T3-L1 adipocytes (ESM Fig. 4).

### Rab3B interaction with Noc2 in adipocytes

Rab3 effectors are known to interact with Munc18 and soluble *N*-ethylmaleimide attachment protein receptor (SNARE) proteins in many vesicle secretory processes [23]. Therefore, we investigated whether known Rab3 effectors associate with Rab3B in an insulin-dependent manner in adipocytes.

**Fig. 5:**
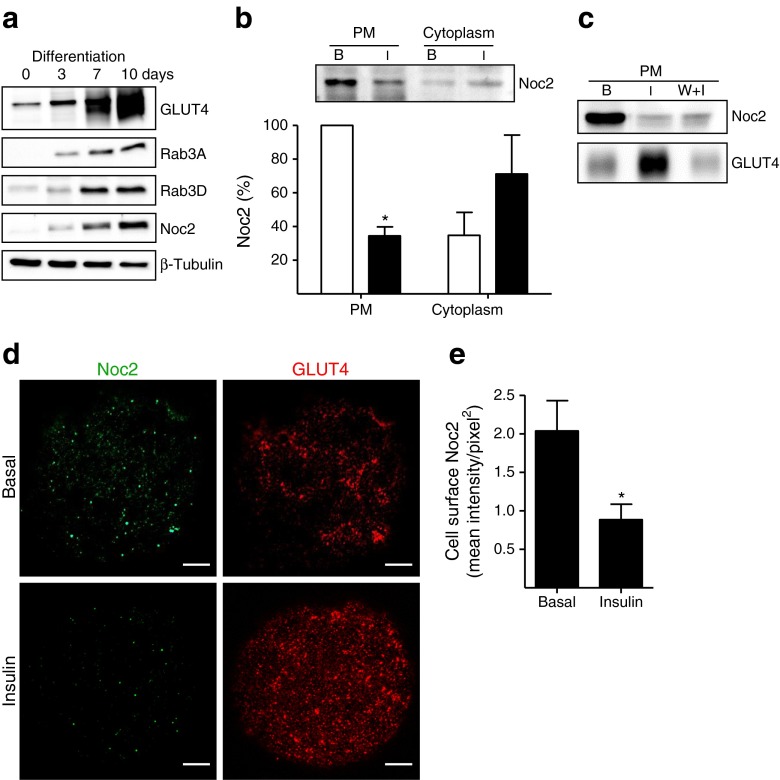
Noc2 is an insulin-responsive Rab3 effector that localises to PMs in rat adipocytes. (**a**) 3T3-L1 fibroblasts and differentiating adipocytes were lysed and immunoblotted for Rab3A, Rab3D, Noc2, GLUT4 and β-tubulin. Representative immunoblots from three independent experiments. (**b**) Subcellular distribution of Noc2 in rat adipocytes. Data are means ± SEM from five independent experiments. *p < 0.05 vs basal. B, basal; I, insulin. White bars, basal; black bars, insulin. (**c**) Effect of wortmannin (W) on insulin-stimulated Noc2 dissociation from PMs. Representative immunoblots from two experiments. (**d**) Noc2 and GLUT4 distribution at the cell surface of rat adipocytes. Confocal images are from single adipose cells representative of the cell populations from at least three separate experiments. Scale bar, 10 μm. (**e**) Comparison of Noc2 immunofluorescent intensity at the cell surface of rat adipocytes. Data are mean intensity/area ± SEM from three independent experiments. *p < 0.05 vs basal. B, basal; I, insulin

The Rab3 effector Noc2 was expressed in insulin-sensitive tissues at the mRNA level (ESM Fig. 5). Noc2 protein expression was found to increase in 3T3-L1 cells during differentiation to adipocytes and occurred in parallel with a corresponding increase in GLUT4 and both Rab3A and Rab3D (Fig. 5a). Noc2 was associated with PM fractions from basal rat adipose cells, was markedly reduced in PM fractions from insulin-treated cells and this occurred concomitantly with an increase in cytoplasmic Noc2 (Fig. 5b). The insulin-stimulated loss of PM Noc2 was not inhibited by wortmannin (Fig. 5c), consistent with the lack of wortmannin inhibition of insulin-stimulated Rab3 activation (Fig. 2c, d). Consistent with these observations, confocal microscopy in the surface-plane of insulin-stimulated rat adipocytes showed reciprocal downregulation of surface endogenous Noc2 and upregulation of surface endogenous GLUT4 (Fig. 5d, e).

**Fig. 6:**
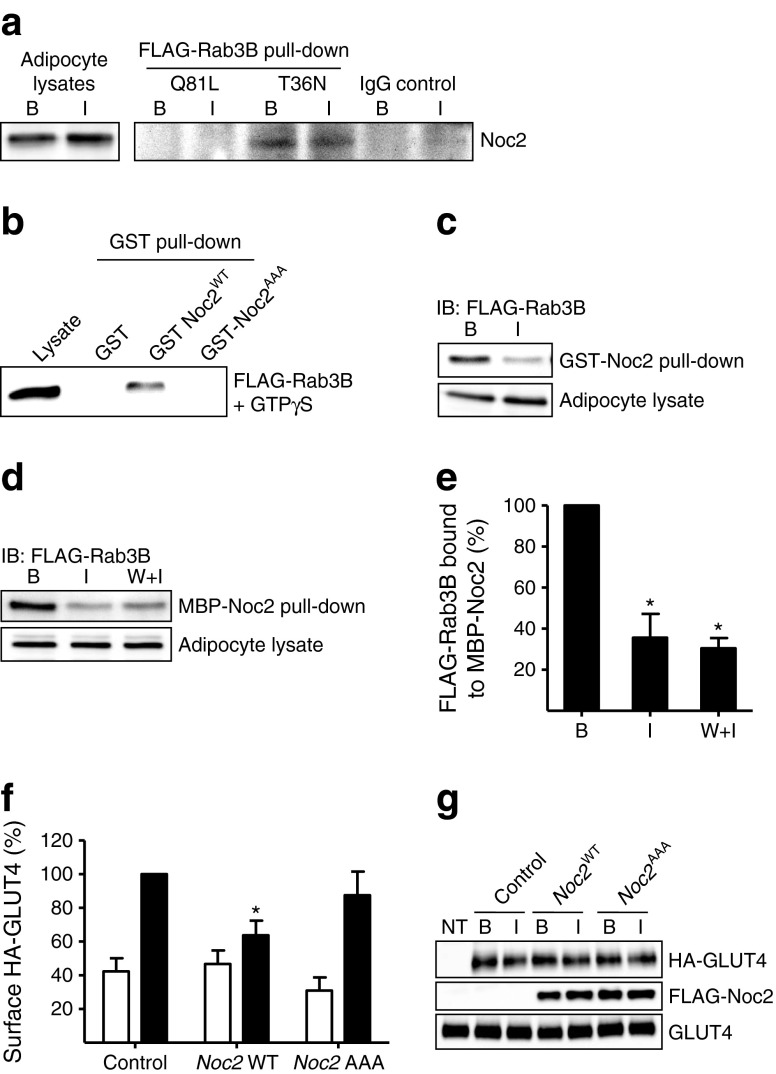
Noc2 is a negative regulator of Rab3B and GLUT4 translocation. (**a**) FLAG-Rab3B^T36N^ and FLAG-Rab3B^Q81L^ recombinant proteins were immobilised on anti-FLAG-antibody–agarose beads and incubated with cell lysate from basal (B) or insulin-stimulated (I) rat adipocytes. Interacting proteins were eluted and immunoblotted for Noc2. A representative immunoblot from three independent experiments is shown. (**b**) FLAG-Rab3B was expressed in rat adipocytes and loaded with GTPγS and then binding to WT Noc2 (GST-Noc2^WT^) and the Rab-domain-deficient mutant (GST-Noc2^AAA^) was examined. A representative immunoblot from three independent experiments is shown. (**c**) Pull-down of FLAG-Rab3B from lysates of basal and insulin-stimulated cells with GST-Noc2^WT^ bound to glutathione beads. Bound FLAG-Rab3B was detected by immunoblotting with anti-FLAG antibody (IB). Representative immunoblots from two independent experiments are shown. (**d**) Identical experiment as in (**c**) performed with MBP-Noc2. W + I, 200 nmol/l wortmannin before insulin stimulation. (**e**) Quantification of the data in (**d**). Data are means ± SEM from three independent experiments. *p < 0.05 vs basal. (**f**) Effect of overexpression of WT Noc2 on GLUT4 translocation. WT Noc2 (FLAG-*Noc2* ^WT^) and the Rab3-binding-deficient mutant (FLAG-*Noc2* ^AAA^) were co-transfected with HA-*Glut4* cDNA in rat adipocytes and the surface HA signal was monitored. Data are means ± SEM from four independent experiments. *p < 0.05 vs HA-*Glut4*-only control. White bars, basal; black bars, insulin. (**g**) Immunoblots of expression of HA-GLUT4, FLAG-Noc2^WT^ and FLAG-Noc2^AAA^ used in the experiments in (**f**). NT, non-transfected cells; B, basal; I, insulin

To examine the interaction of Noc2 and Rab3B, FLAG-Rab3B^T36N^ and FLAG-Rab3B^Q81L^ recombinant proteins were bound to anti-FLAG-antibody–agarose beads and incubated with lysate from basal or insulin-stimulated rat adipocytes (Fig. 6a). Binding of Noc2 occurred only to the GDP-bound mutant FLAG-Rab3B^T36N^ and not to the constitutively active FLAG-Rab3B^Q81L^. The interaction between Rab3B and Noc2 was confirmed by reciprocal pull-down experiments. GST-Noc2 WT and Rab3-binding-deficient mutant GST-Noc2^AAA^ [24] were compared (Fig. 6b) for their ability to bind FLAG-Rab3B in the presence of GTPγS. Only WT Noc2 bound FLAG-Rab3B.

The ability of FLAG-Rab3B from adipocyte lysates to bind to Noc2 was assessed using an immobilised MBP-Noc2 construct (Fig. 6d, e) and a GST-Noc2 construct (Fig. 6c). There was much less interaction when the lysates were prepared from insulin-stimulated cells and this effect was not sensitive to wortmannin (consistent with the absence of a wortmannin inhibitory effect on insulin-stimulated Noc2 dissociation from the PM). Since the GDP-bound mutant of Rab3 preferentially binds Noc2 (Fig. 6a), these observations are consistent with the possibility that insulin activation leads to increased conversion of lysate GDP-Rab3 to GTP-Rab3 with an associated reduction of complex formation with Noc2.

To determine whether the Rab3B–Noc2 interaction can influence GLUT4 translocation we measured HA-GLUT4 translocation in basal and insulin-treated adipose cells in the presence of WT and *Noc2*^AAA^ constructs that were expressed at similar levels (Fig. 6g). Only the wild type (not the inactive *Noc2*^AAA^ mutant) inhibited GLUT4 translocation (Fig. 6f). These data suggest that the negative regulatory effects of Noc2 on Rab3B function impinge upon the GLUT4 translocation process.

## Discussion

A new GTP photolabel that can be used in conjunction with a mass spectrometry-based proteomics approach led us to identify Rab3B as an insulin-activated GTPase in rat adipocytes and a novel component of the GLUT4 translocation process. Focusing on the mechanism for Rab3-dependent regulation led us to discover that Noc2 is a negative modulator of Rab3 activity and GLUT4 translocation and that it dissociates from the PM upon insulin activation and GTP loading of Rab3. Therefore we propose a new mechanism by which the final stages of GSV translocation are controlled in insulin target cells. The combined use of the new GTP photolabel and proteomics is a strength of the study. However, use of this reagent has efficiency limitations. It can be insensitive to G-proteins that are either expressed at low levels or insulin activated in a small subpopulation of the total cellular pool.

Variations occur in the prevalence of Rab3 isoforms in insulin-sensitive tissues, such as muscle and heart, and other systems in which regulated vesicular secretory processes occur, including brain [25] (ESM Fig. 3a) and pancreas [26]. This suggests that there is significant functional redundancy between Rab3 isoforms. Rab3A and D have been reported to be present in adipocytes but studies on their relevance to GLUT4 traffic have yielded conflicting results [20–22]. Functional redundancy among Rab3 isoforms may account for our observations from Rab3 knockdown experiments carried out in mouse 3T3-L1 cells. Rab3A knockdown gave rise to a relatively small but significant decrease in insulin-stimulated glucose transport in this system, but it was necessary to knock down Rab3A, Rab3D and Rab3B to achieve a substantial (35% decrease) reduction in the insulin-stimulated glucose transport. Experiments directed more specifically at determining effects of Rab3 knockdown on GLUT4 translocation revealed that the insulin-stimulated increase in cell-surface GLUT4 was inhibited by over 80%. The discrepancy between the levels of inhibition of 2-deoxy-d-glucose uptake and the inhibition of GLUT4 translocation is most likely due to variable and incomplete knockdown of Rab3 isoforms. However, GLUT1 also contributes to insulin-stimulated glucose uptake in these cells [27]. GLUT1 is translocated via a route that is distinct from that associated with GLUT4 [28, 29] and may be less dependent on Rab3. These data are consistent with a role for Rab3 in regulating GLUT4 translocation and support the data obtained using the rat adipocyte system where Rab3B is most abundant and important.

One of the most important steps in regulation of GLUT4 traffic, as it responds to insulin stimulation of cells, involves stimulation of exocytosis [28, 30] and recent experiments have led to consideration of the docking and fusion of the vesicles with the PM as the key regulated sub-step in exocytosis [4, 31–33]. Vesicle docking and fusion have been identified as Rab3-modulated steps in many studies of vesicle secretory processes [6] and it therefore seems possible that regulated GLUT4 vesicle exocytosis may be similarly dependent on Rab3 for full functionality. Consistent with this we find that constitutively active Rab3B raises GLUT4 at the cell surface of basal adipose cells to a level equal to that observed with insulin stimulation. By contrast, the dominant negative Rab3B mutant inhibits insulin-stimulated GLUT4 translocation.

The typical Rab3 effectors are synaptotagmin-like proteins (Slps) which contain two C2 domains for membrane attachment, and a Rab-interacting domain [34]. Slps include rabphilin 3A and granuphilin. Granuphilin was present in adipocytes but we found no evidence that its cellular distribution was altered by insulin. Other Rab3 effectors that lack the C2 domains have been described [34]. Among them is Noc2 (gene symbol *Rph3al*), a negative regulator of exocytosis [35]. Interestingly, the genes coding for Noc2 and Doc2B (a known GLUT4 regulatory protein [36, 37]) are adjacent on chromosome 10 in rats. Separation of function for two ancient domains (Rab-interacting domain [*Rph3al*] and C2 domains [*Doc2b*]) in a single gene of the rabphilin type may have occurred, possibly to allow hormone regulation of membrane docking and fusion. Our data suggest that Noc2 has a negative regulatory role in insulin target cells. Consistent with this, we found that Noc2 bound to an inactive Rab3B construct (Rab3B^T36N^) but not to a constitutively active Rab3 construct (Rab3B^Q81L^). The interaction of Noc2 with Rab3B was confirmed by expressing a recombinant Noc2 and an inactive mutant Noc2^AAA^. Rab3B only bound the former. Furthermore, the interaction of Noc2 with Rab3B was reduced in insulin-treated cells suggesting that Noc2 may quench the availability of Rab3 by selectively binding the inactive GDP form of the protein.

**Fig. 7:**
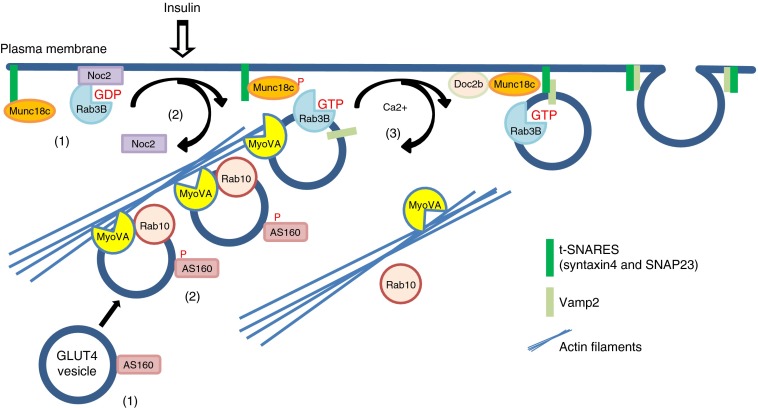
Rab3 modulation of the final stages of insulin-regulated GLUT4 vesicle exocytosis. In the basal state AS160 is active and maintains Rab10 in an inactive state. Noc2 is bound to PMs and maintains Rab3B in its GDP/inactive state (1). Upon insulin stimulation AS160 is phosphorylated, Rab10 is activated and interacts with MyoVA to facilitate the movement of GLUT4 vesicles to the PM. Insulin stimulation leads to Rab3B GTP loading and an associated Noc2 release from the PM (2). This allows Rab3B to interact with GLUT4 vesicles, which then dissociate from Rab10–MyoVA-associated actin and engage with SNAREs (3)

We found that Noc2 is displaced from the PM and is released into the cytoplasm of insulin-treated adipocytes, suggesting an inhibitory role for PM Noc2 that is relieved by insulin-induced loss of Noc2 to the cytoplasm fraction. This inhibitory effect is consistent with observed inhibitory effects of the WT but not the mutant form of Noc2 on GLUT4 translocation. These findings have led us to propose a role for Rab3–Noc 2 dissociation in the modulation of GLUT4 vesicle exocytosis at a vesicle docking and fusion step (Fig. 7). This modulation would occur at a step beyond the delivery of GSVs to the PM, which occurs via a Rab10- and myosin-VA (MyoVA)-dependent step. Chen et al [4] have recently demonstrated that MyoVA and Rab10 co-localise to GSVs, which are recruited from the intracellular reserve compartment to the total internal reflection fluorescence zone (200 nm of the PM) in 3T3-L1 cells. However, MyoVA also binds Rab3 isoforms [38]. We therefore propose that the TBC1D4/GTP-loaded-Rab10–MyoVA complex delivers GSVs to an insulin-activated Rab3 complex. As the interaction between Rab3 and Noc2 is insulin-dependent but not wortmannin inhibitable, we further propose that PI 3-kinase-independent insulin action leads to release of Rab3B from Noc2 and then GSVs transfer from MyoVA and actin filaments to interact with Munc18c, Doc2B and the SNARE fusion molecules [39–42] that facilitate the final stages in GLUT4 exocytosis (Fig. 7).

The novel discovery that Rab3 and its effector Noc2 modulate GLUT4 translocation identifies a key, previously unidentified, step coupling insulin action to GSV traffic. This system resembles regulated exocytosis of insulin vesicles in pancreatic islet cells. Insulin release also involves similar SNAREs and Munc18c and Rab3 components [23, 41]. Therefore, impairments at this site in vesicle exocytosis in the pancreas and peripheral insulin-sensitive tissues may concurrently lead to linked effects on glucose homeostasis through changes in insulin secretion and GLUT4 trafficking. This concurrent effect may compound pathophysiological effects due to a more sequential peripheral insulin resistance that is followed by hyperinsulinaemia and then beta cell loss.

## Electronic supplementary material

ESM Methods (PDF 224 kb)

ESM Fig. 1 (PDF 86 kb)

ESM Fig. 2 (PDF 89 kb)

ESM Fig. 3 (PDF 86 kb)

ESM Fig. 4 (PDF 114 kb)

ESM Fig. 5 (PDF 90 kb)

ESM Table 1 (PDF 53 kb)

ESM Table 2 (PDF 63 kb)

## Abbreviations

ATB: Azitrifluoroethylbenzoyl
Bio-ATB-BGPA: Bio-azitrifluoroethylbenzoyl-bis-glucose-propyl-2-amine
GSV: GLUT4 storage vesicle
MBP: Maltose binding protein
MyoVA: Myosin-VA
PI 3: Phosphoinositide 3
PM: Plasma membrane
Rab-GAP: Rab GTPase activating protein
Rab-GEF: Rab guanosine exchange factor
siRNA: Small interfering RNA
SNARE: Soluble *N*-ethylmaleimide attachment protein receptor
WT: Wild-type
Noc2^AAA^: Rab-binding deficient mutant *Noc2*^W154A, F155A, Y156A^
