# Supplementary material for: Insulin regulates Rab3–Noc2 complex dissociation to promote GLUT4 translocation in rat adipocytes
Source: Diabetologia. 2015 May 30;58(8):1877–86. doi: 10.1007/s00125-015-3627-3 (PMC4499112; doi:10.1007/s00125-015-3627-3)
Supplement: Supplementary file 1 — (PDF 224 kb) [file 125_2015_3627_MOESM1_ESM.pdf]

Electronic supplementary materials (ESM) Methods

Bio-ATB-GTP photolabel synthesis

2-[2-[2-[2-(2-*tert*-Butoxycarbonylaminoethoxy)ethoxy]ethoxy]-4-[3-(trifluoromethyl)-3*H*-diazirin-3yl] benzoic acid [1] was coupled with Biotinylcaproate and then converted to the NHS ester [2]. 2 µmoles of this material in 20 µl of DMF was added to a solution of 0.9 µmoles 2'/3'-O-(2-Aminoethyl-carbamoyl)-Guanosine-5'-triphosphate [3] in 90 µl water followed by 1.2 µmoles of N,N-diisopropylethylamine base in 4.3 µl DMF. The reaction mix was left for approximately 18 h at RT. The solvents were evaporated under a nitrogen stream and 200 µl of water added. Non-soluble material was removed by centrifugation in a microfuge for 2 min. The clear supernatant was added to an activated 1 ml Sep-Pak column (Waters, Milford, MA, USA) and eluted sequentially with 3 aliquots of 0.4 ml water followed by 3 aliquots of 30% ethanol/water and then 3 aliquots of 60% ethanol/water. The fractions were monitored by thin layer chromatography and then the 30% ethanol fractions were combined and concentrated to give product that was usually 90% pure as determined by HPLC. Further purification was obtained by preparative HPLC using a gradient (0-60%) of triethylamine acetate and acetonitrile. The product was obtained in yields of approximately 30%. The Bio-ATB-GTP structure (ESM Fig. 1a) was confirmed by mass spectroscopy (Bruker, Coventry, UK, MicroTOF, +ve ion). Predicted for C44H63F3N13O22P3S: 1307.3096; Found: 1308.3169 (MH+).

Photolabelling of membranes with Bio-ATB-GTP

300 µg of total membranes were incubated in a 96 well plate in a final volume of 100 µl of HES buffer containing 2 mmol/l EDTA. 40 µmol/l final concentration of Bio-ATB-GTP was added to the membranes and incubated for 30 min on ice with occasional mixing. At the end of the incubation time, 5 mmol/l final concentration of $MgCl_2$ was added to the membrane mixture and incubated for a further 5 min. The membranes were irradiated for 2 x 1 min in a Rayonet UV chamber at λ = 350 nm. Membranes were washed from excess un-bound Bio-ATB-GTP by diluting the membranes to 3 ml and centrifuging at 100,000 rpm for 30 min in TLA100.3 rotor (Beckman, High Wycombe, UK). The recovered pellet of membranes was resuspended in RIPA buffer (50 mmol/l Tris pH 7.4, 150 mmol/l NaCl, 0.5% sodium deoxycholate, 0.1% sodium dodecyl sulfate, 0.1% NP-40) supplemented with protease inhibitors and left to solubilize for 1 h at 4°C with end to end rotation. At the end of the incubation time insoluble material was discarded by centrifuging at 17 000 g for 20 min at 4°C and the supernatant containing the solubilized proteins was used in further experiments.

Streptavidin precipitation of biotinylated proteins.

Membrane or cell lysates containing biotinylated proteins were incubated with 60 µl of streptavidin agarose slurry (Thermo Scientific, Rockford, IL, USA)) overnight at 4°C with end to end rotation. The next day the beads were washed twice with lysis buffer, twice with 1 to 10 diluted lysis buffer in PBS and twice with PBS. Biotinylated proteins were then eluted from the beads by incubating twice in SDS-sample buffer for 20 min at 95°C. The two elutions were pooled together and the protein separated by SDS-PAGE. After electrotransfer to nitrocellulose membranes, proteins of interested were detected by immunoblotting with the relevant antibody as indicated in the figure legends.

2-D gel separation of Bio-ATB-GTP labelled total membranes

After photolabelling with the Bio-ATB-GTP analogue 200 µg of membranes were spun down to remove free label. The resulting pellets were resuspended in 250 µl BioRad 2D gel Sample buffer, containing 1% tributylphosphine (TBP) reducing agent (BioRad, Hercules, CA, USA)

and 2% IPG buffer (3-10) (GE Healthcare, Little Chalfont, UK). Samples were sonicated and spun at 13 000 rpm for 5 min to remove unsoluble material. All the following steps for the isoelectric focussing (IEF) are taken from 2-D gel electrophoresis Handbook (GE Healthcare). Samples were loaded on 13 cm 3-10 NL Immobiline DryStrip gel (GE Healthcare) at room temperature overnight using the rehydration method. The next day the Immobiline DryStrip gel strips were focused on a Multiphore II Electrophoresis system using the following parameters: Gradient mode, Step 1 300 V for 0.01 h, Step 2 3500 V for 1.5 h and Step 3 3500 V for 4.5 h. For all stages, the current limit was set to 10 mA, and the power limit to 5 W. The temperature of the Multiphor was maintained at 17°C using a recycling thermostatic water bath. At the end of the IEF, the Immobiline DryStrip gel strips were incubated in SDS equilibration buffer (75 mmol/l Tris pH 8.8, 6 mol/l Urea, 29.3% (v/v) glycerol, 2% SDS, 0.002% Bromophenol Blue) supplemented with 10 mg/ml DTT, followed by incubation in SDS equilibration buffer supplemented with 25 mg/ml iodoacetamide. The strips were, loaded on a 15% Tris-Glycine Gel and ran in a Protean II xi BioRad cell. At the end of the electrophoresis run the gels were partially electrotransfered to nitrocellulose membrane by semi-dry transfer for 10 min at 150 mA for one gel. The gel was then washed and stained with PageBlue Protein Staining solution (Thermo Scientific), or with SyproRuby gel stain (Life Technologies, Paisley, UK). The nitrocellulose membrane was analysed for presence of biotinylated protein by incubation with ExtrAvidin^{TM}-HRP (1/25000 dilution) (Sigma, St Louis, MO, USA).

Proteomic analysis
Gel spots of interested were excised from the 2-D gels, trypsin digested and analysed by MALDI TOF, or nano-LC-QTOF MS/MS at the proteomic facility at the University of the West of England, Bristol, United Kingdom.

Antibodies
The following antibodies were used in this study: rabbit polyclonal GLUT4 antibody was raised against a GLUT4 C-terminal peptide [4]; mouse anti HA antibody (Clone 16B12) (Covance, Emeryville, CA, USA); anti Rab11 mouse monoclonal antibodies (BD Biosciences, Oxford, UK); mouse anti Rab3, Rab3A and rabbit anti Rab3D antibodies (Synaptic Systems, Göttingen, Germany); mouse anti Rab3 and Rab3B antibodies (Abcam, Cambridge, UK); mouse monoclonal anti FLAG antibody (Sigma); rabbit anti Noc2 antibody (ProteinTech, Chicago, IL, USA); mouse IgG secondary antibody β-galactosidase conjugate was from SouthernBiotech (Birmingham, AL, USA).

DNA constructs
pCis2 *HA-GLUT4* was a gift from Dr. Samuel Cushman and has been described previously. *Rab3B* was amplified from a rat brain cDNA library and initially cloned in pET28a(+) bacterial expression vector. Subsequently, this clone was used for all subcloning procedures in pcDNA3.1 and in p3xFLAG-CMV10 vectors. The constitutively active mutant Rab3B^{Q81L} and the inactive mutant Rab3B^{T36N} were generated using the QuikChange™ Site-Directed Mutagenesis Kit protocol (Stratagene, La Jolla, CA, USA). Noc2 (*Rph3al*) was amplified from rat heart cDNA library and cloned in pGEX4T-1 bacterial expression vector. This clone was subsequently used for all subcloning procedures in pMAL-c2 and p3xFLAG-CMV10 expression vectors. The Rab-binding deficient mutant Noc2^{AAA} (W154A, F155A, Y156A) was generated using the QuikChange™ Site-Directed Mutagenesis Kit protocol (Stratagene).

Quantification of Rab3 isoform and Noc2 mRNA levels

Total RNA was extracted from rat brain, skeletal muscles, heart, and epididymal adipose tissue with TriPure isolation reagent (Roche Diagnostics, Mannheim, Germany) according to the manufacturer's instructions. Total RNA from 3T3-L1 adipocytes was extracted using the SV Total RNA isolation system (Promega, Madison, WI, USA) according the manufacturer's instructions. 500 ng of total RNA, treated with DNaseI, was reverse transcribed to cDNA using Superscript III First-Strand Synthesis Supermix for qRT-PCR kit (Life Technologies). qRT-PCR was performed with the StepOnePlus™ Real-Time PCR System (Life Technologies) using iTaq™ SYBR® Green supermix with ROX (BioRad) according to the manufacturer's instructions. 5 ng adipose cDNA and 500 nmol/l (final concentration) primers were used in the final qRT-PCR reaction. Primers were designed using Primer3 software (http://frodo.wi.mit.edu/primer3;[5] and synthesised by Sigma-Aldrich (ESM Table 1). Primers were validated against a standard curve of varying amounts of brain cDNA (for Rab3A, B and C), testis cDNA (for Noc2) and adipocyte cDNA (for Rab3B and D), tissues where the Rab3 isoforms and Noc2 are known to be present. The comparative CT method was used to quantify the relative expression of *Rab3* and *Rph3al* in rat tissues and 3T3-L1 adipocytes.

Expression and purification of recombinant proteins

GST-fusion proteins were expressed in *E. Coli* strain BL21 by induction with 0.1 mmol/l IPTG for 3 h at 21°C. The recombinant proteins were purified on a glutathione-sepharose column and the eluted proteins dialysed against PBS.

MBP-*Noc2* cDNA was transformed into *E. coli* strain TB1. Protein expression was initiated by induction with 0.1 mmol/l IPTG for 3 h at 21°C. Bacterial lysate was clarified by centrifugation and the resulting supernatant containing the MBP-Noc2 was purified by attachment to amylose resin. The eluted proteins were dialysed against 20mmol/l Tris (pH 7.6), 200 mmol/l NaCl and stored frozen at -70 ºC for further use.

FLAG-*Rab3B*^{Q81L} or FLAG- *Rab3B*^{T36N} were transfected into the human embryonic kidney (HEK) 293T cell line using a calcium phosphate transfection method and after 48 h expression, the cells were lysed and the lysate was clarified by centrifugation. The resulting supernatant containing the FLAG tagged constructs was stored frozen at -70 ºC for further use. When required, the recombinant protein was purified by immunoprecipitation with anti-FLAG antibody agarose conjugate (Sigma).

Isolation of primary rat adipocytes

Adipose cells from epididymal fat pads of male Wistar rats (in-house bred colony, fed on a standard chow diet), weighing 180–200 g, were prepared by collagenase digestion as described previously [6]. Cells were maintained at 37ºC in Krebs-Ringer-HEPES (KRH) buffer (140 mmol/l NaCl, 4.7 mmol/l KCl, 2.5 mmol/l $CaCl_2$, 1.25 mmol/l $MgSO_4$, 2.5 mmol/l NaH_2PO_4, 10 mmol/l HEPES, (pH 7.4)) with 1% (w/v) bovine serum albumin (BSA) and 200 nmol/l adenosine. Before transfection, the cells were washed twice with DMEM supplemented with 200 nmol/l adenosine and brought to a 50% cytocrit.

Adipocyte transfection

Rat adipocytes were electroporated with pCis2 *HA-GLUT4* alone or together with pcDNA3.1-*Rab3B* WT, Q81L or T36N or p3xFLAG- *Rab3B* WT, Q81L or T36N or p3xFLAG-*Noc2* WT or AAA according to the method described by Al-Hasani et al. [7]. Briefly, 200 µl of 50% cytocrit rat adipocytes were added to 200 µl of DMEM containing 100 µg of carrier DNA (hearing sperm DNA, Promega) and 0.1 µg of pCis2 *HA-GLUT4* cDNA alone or together with 0.4 µg of *Rab3B* or *Noc2* construct cDNA. The amounts of *HA-*

GLUT4 and *Rab3B* construct cDNA were optimised as described [7]. Electroporation was carried out using the BioRad Gene pulser with a capacitance extender attached in 0.4 cm gap-width cuvettes (BioRad). Each cuvette was electroporated once at 400 V, 500 μF. Cells from 4 to 5 cuvettes were pooled together, washed once with DMEM supplemented with 200 nmol/l adenosine and then resuspended in DMEM supplemented with 3.5 % BSA and 200 nmol/l adenosine. The transfected adipocytes were incubated for 18 h at 37ºC. After washing in KRH buffer supplemented with 1% BSA and 200 nmol/l adenosine cells were left unstimulated or stimulated with 100 nmol/l insulin for 20 min at 37ºC. Using the above described electroporation conditions we obtained 15% to 20% transfection efficiency as estimated by examining the cells by fluorescent microscopy and recording GFP positive cells (data not shown).

Determination of surface HA-GLUT4

Rat adipocytes cell surface HA-GLUT4 was detected following the method described in [8]. In brief, transfected rat adipocytes, left unstimulated or stimulated with 100 nmol/l insulin, were incubated in presence of 2 mmol/l KCN for 3 min to stop GLUT4 recycling. Cells were then incubated with 1 μg/ml anti-HA antibody in KRH buffer supplemented with 5% BSA and 200 nmol adenosine for 1 h at room temperature with occasional mixing. After three washes in KRH buffer supplemented with 5% BSA and 200 nmol/l adenosine, adipocytes were incubated with 1 μg/ml anti-mouse IgG secondary antibody β-galactosidase conjugate. Cells were then washed in KRH buffer supplemented with 200 nmol/l adenosine 4 times and 10 μl of cell suspension were added in quadruplicate in black 96 well plates (Fluotrac 200, Greiner, Stonehouse, UK). Fluorescein digalactosidase (FDG) at a final concentration of 0.1 mmol/l in KRH buffer was added to each well. The rates of fluorescence generated per mg protein were then determined from measurements (for an hour at 15 sec intervals) in a PHERAstar FS fluorescent plate reader (BMG LABTECH, Ortenberg, Germany) at 520 nm. An aliquot of cells from each condition was analyzed by immunoblotting to assess and normalize for the level of expression of HA-GLUT4 cDNA.

Rat adipose cell fractionation

Freshly isolated adipocytes or adipocytes transfected by electroporation were left untreated or were treated with 20 nmol/l insulin or 100 nmol/l insulin for 20 min respectively. Wortmannin treatment was performed by incubating the cells with 200 nmol/l wortmannin for 10 min prior to the insulin stimulation. At the end of the treatment, cells were washed once with KRH buffer and then once with HEPES-EDTA-Sucrose (HES) buffer (20 mmol/l HEPES pH 7.2, 1 mmol/l EDTA, 255 mmol/l sucrose) cooled to 18°C. The cells were then homogenized with ten strokes of a Potter-Elvehjem homogenizer and fat cake and cell debris were removed by spinning the homogenate for 1 min at 1000 g at 4°C.

To obtain total adipocyte membranes the resulting homogenate was spun at 100 000 rpm for 30 min in TLA100.3 rotor (Beckman) at 4°C. The pellet of total adipocyte membrane was resuspended in HES buffer, homogenized again to disrupt unbroken cells and the membranes were pelleted down by centrifugation at 100 000 rpm for 30 min in TLA100.3 rotor (Beckman).

Adipocytes subfractionation was performed according to the method described by Simpson *et al.* [9].

GLUT4 containing vesicles were isolated following the protocol described previously [10]. Whole cell adipocyte lysate was obtained by washing the cells after treatment with KRH buffer twice and then lysing the cells for 20 min at 18°C in the desired lysis buffer as indicated in the individual protocols. Lysates were cleared by centrifugation at 17 000 g at 4°C. The resulting supernatant was used in the subsequent pull-down experiments.

FLAG-Rab3B pull-down of adipose cell interacting protein.
FLAG-Rab3B^{Q81L} or FLAG- Rab3B^{T36N} overexpressed in HEK293T cells were pre-bound to anti-FLAG antibody conjugated agarose beads. After extensive washes to remove non-specifically bound proteins the beads were incubated for 2 h with whole cell adipocyte lysate prepared from control or insulin stimulated cells lysed in Triton X-100 lysis buffer (20 mmol/l HEPES pH 7.5, 100 mmol/l NaCl, 5 mmol/l $MgCl_2$, 1% (v/v) Triton X-100) supplemented with protease and phosphatase inhibitors. At the end of the incubation time the beads were extensively washed and Rab3B interacting proteins were eluted with high salt elution buffer (20 mmol/l HEPES pH7.5, 1.5 mol/l NaCl, 20 mmol/l EDTA, 0.1% Triton X-100). Eluted samples were desalted before running on SDS-PAGE. After electrotransfer, proteins were analysed by immunoblotting with relevant antibodies.

GST-Noc2 and MBP-Noc2 pull-down of FLAG-Rab3B
50 µg GST-Noc2 or 30 µg MBP-Noc2 wild type (WT) or AAA were bound to glutathione or amylose beads respectively. Rat adipocytes transfected with FLAG-Rab3B WT were left untreated or treated with 100 nmol/l insulin for 20 min. Wortmannin inhibition was performed by incubating the cells with 200 nmol/l wortmannin for 10 min before the insulin stimulation. After one wash with KRH buffer cells were lysed for 20 min at 18°C in 10 mmol/l Tris-HCl pH 7.5, 100 mmol/l NaCl, 5% glycerol, 1% (v/v) Triton X-100 supplemented with protease and phosphatase inhibitors. The resulting adipocyte cell lysate containing overexpressed FLAG-Rab3B was incubated for 1 h at 4°C with GST-Noc2 or MBP-Noc2 beads. After extensive washes with lysis buffer, proteins bound to the GST-Noc2 were eluted with SDS sample buffer. Proteins bound to the MBP- Noc2 were eluted with maltose elution buffer (20 mmol/l Tris-HCl pH 7.5, 200 mmol/l NaCl, 5 mmol/l $MgCl_2$, 40 mmol/l maltose). Eluted protein samples were separated by SDS-PAGE, electrotransferred and immunoblotted with anti-FLAG antibody.
To compare the binding of FLAG-Rab3B to GST-Noc2 WT and GST-Noc2^{AAA} the basal adipocyte lysate containing the overexpressed FLAG-Rab3B was incubated with 1 mmol/l GTPγS for 10 min at 37°C. The GTPγS loading was stabilised by the addition of a final concentration of 10 mmol/l $MgCl_2$.

Immunofluorescent microscopy
Rat adipocytes were washed with KRH buffer and then with PBS before fixing in 4% paraformaldehyde for 20 min at room temperature (RT). Cell were then washed with PBS, quenched with 100 mmol/l glycine in PBS and treated with permeabilisation buffer (0.1% saponin, 1% (w/v) BSA, 3% (v/v) goat serum in PBS) for 45 min at RT. Rabbit anti-Noc2 antibody (1/100 dilution) and mouse anti-GLUT4 antibody (clone 1F8) (1/200 dilution) were added to the cells and incubated overnight. After extensive washes cells were incubated for 2 h with AlexaFluor tagged secondary antibodies (Life Technologies) as indicated in the Figure legend. After a final wash step in permeabilisation buffer, cells were mounted onto a glass coverslip with Vectashield mounting medium (Vector Laboratories Burlingame, CA, USA). Confocal microscopy was performed on a Zeiss LSM 510 META microscope with 63x 1.4 NA oil-immersion objectives and with dual laser excitation at 458–488 and 543 nm. Images 2048x2048 were acquired with identical settings. TIFF files were exported and processed in Photoshop to adjust to the same dynamic range. The mean intensity per square pixel in the green channel (Noc2 signal) of individual cells was measured with the Measure tool in the ImageJ National Institute of Health software (http://imagej.nih.gov/ij/). Typically at least 5 individual cells were measured per condition and the experiment was repeated 3 times.

3T3-L1 adipocyte culture and siRNA silencing.

3T3-L1 fibroblasts were obtained from the American Type Culture Collection (Manassas, VA, USA), and were cultured in DMEM and differentiated to adipocytes by treatment with insulin, dexamethasone and isobutylmethylxanthine as described previously [11]. 3T3-L1 adipocytes at day 3 or 4 of differentiation were used for siRNA transfection according to the protocol described by Kilroy et al. [12]. Briefly, 3T3-L1 adipocytes at day 3 of differentiation were lifted from one 10 cm dish by trypsin digestion and resuspended in DMEM supplemented with 10% FBS without antibiotic at a density of $5.8x10^5$ cells/ml. The siRNA mixture with transfection reagent was prepared in 24 well plates as follows. 25 µl of smart pool siRNA was mixed with 25 µl of OptiMEM in each well. After 5 min incubation at room temperature, 50 µl OptiMEM containing 2.8 µl of DharmaFECT1 transfection reagent was added and incubated for a further 20 min to allow the siRNA complex to form. At the end of the incubation time 0.5 ml of resuspended 3T3-L1 adipocytes were added to each well and the cells were incubated for 24 hours before changing the medium to DMEM with 10% FBS. The final concentration of siRNA was 100 nmol/l and of DharmaFECT1 - 1.4 µl/cm^2. The seeding density was $1.16x10^5$ cells/cm^2. All ON-TARGETplus SMART pool siRNA preparations as well as the DharmaFECT1 reagent were from Thermo scientific: mouse *Rab3A* (L-060407-01), mouse *Rab3B* (L-059841-01), mouse *Rab3D* (L-040852-01), Non-targeting pool (D-0018100-10). Cells were used for experiments 3 or 4 days post transfection. Non-targeting siRNA was added to the same final concentration as the targeting Rab3 siRNAs.

Measurement of 2-deoxy-D-glucose transport in 3T3-L1 adipocytes

24 h prior to the measurement of glucose transport, the cells were given fresh DMEM-FCS. On the day of the experiment the cells were incubated with serum-free DMEM for 2 h at 37° C. After this incubation period, the cells were washed three times with KRH buffer and incubated for 30 min in either the absence or presence of 100 nmol/l insulin at 37°C. After the thirty minute incubation period, radiolabelled 2-deoxy-D-[2,6-^{3}H]glucose (final concentration 50 µmol/l, 0.1 µCi/well) was added for 5 min, and then the cells were washed four times with ice-cold KRH buffer. Nonspecific 2-deoxy-D-glucose uptake was measured in the presence of 10 µmol/l cytochalasin B. The cells were lysed and the radioactivity was extracted into 0.1 mol/l NaOH. Results were then expressed in pmols of 2-deoxy-D-glucose/min/mg of proteins.

GLUT4 and GLUT1 photolabelling in 3T3-L1 adipocytes.

Cells were serum-starved and insulin-stimulated as described above. At the end of the insulin stimulation the cells were cooled down to 18°C and irradiated for 1 min at 300 nm UV light in a Rayonet photoreactor with 300 µmol/l final concentration Bio-ATB-BGPA photolabel [1]. Cells were then rapidly washed with ice-cold KRH and lysed in 2% Thesit ($C_{12}E_9$) in PBS. The biotinylated transporters were precipitated with Streptavidin agarose as described above.

Immunoblotting image acquisition and processing

All immunoblots where incubated with enhanced chemiluminescent substrate and images where acquired with UVP EpiChemi II Darkroom and LabWorks imaging software (UVP, Cambridge, UK). Semi quantitative analysis of the original images was performed using the LabWorks software. LabWorks software was used to generate 8 bit images using the best fit function, these images were cropped appropriately and then exported as Tiff files.

Statistical analysis

Results were analysed using two-tailed un-paired *t* tests. A p value < 0.05 was considered statistically different.

References

[1] Hashimoto M, Hatanaka Y, Yang J, Dhesi J, Holman GD (2001) Synthesis of biotinylated bis(D-glucose) derivatives for glucose transporter photoaffinity labelling. Carbohydr Res 331: 119-127
[2] Koumanov F, Yang J, Jones AE, Hatanaka Y, Holman GD (1998) Cell-surface biotinylation of GLUT4 using bis-mannose photolabels. Biochem J 330: 1209-1215
[3] Jameson DM, Eccleston JF (1997) [18] Fluorescent nucleotide analogs: Synthesis and applications. In: Ludwig Brand MLJ (ed) Methods Enzymol. Academic Press, pp 363-390
[4] Satoh S, Nishimura H, Clark AE, et al. (1993) Use of bismannose photolabel to elucidate insulin-regulated GLUT4 subcellular trafficking kinetics in rat adipose cells. Evidence that exocytosis is a critical site of hormone action. J Biol Chem 268: 17820-17829
[5] Rozen S, Skaletsky H (2000) Primer3 on the WWW for general users and for biologist programmers. Methods Mol Biol 132: 365-386
[6] Taylor LP, Holman GD (1981) Symmetrical kinetic parameters for 3-O-methyl-D-glucose transport in adipocytes in the presence and in the absence of insulin. Biochim Biophys Acta 642: 325-335
[7] AlHasani H, Kinck CS, Cushman SW (1998) Endocytosis of the glucose transporter GLUT4 is mediated by the GTPase dynamin. J Biol Chem 273: 17504-17510
[8] Koumanov F, Pereira VJ, Whitley PR, Holman GD (2012) GLUT4 traffic through an ESCRT-III-dependent sorting compartment in adipocytes. PLoS One 7: e44141
[9] Simpson IA, Yver DR, Hissin PJ, et al. (1983) Insulin-stimulated translocation of glucose transporters in the isolated rat adipose cells. Characterization of subcellular fractions. Biochim Biophys Acta 763: 393-407
[10] Koumanov F, Richardson JD, Murrow BA, Holman GD (2011) AS160 Phosphotyrosine-binding Domain Constructs Inhibit Insulin-stimulated GLUT4 Vesicle Fusion with the Plasma Membrane. J Biol Chem 286: 16574-16582
[11] Frost SC, Lane MD (1985) Evidence for the involvement of vicinal sulfhydryl groups in insulin-activated hexose transport by 3T3-L1 adipocytes. J Biol Chem 260: 2646-2652
[12] Kilroy G, Burk DH, Floyd ZE (2009) High efficiency lipid-based siRNA transfection of adipocytes in suspension. PLoS One 4: e6940
