# Supplementary material for: Insulin regulates Rab3–Noc2 complex dissociation to promote GLUT4 translocation in rat adipocytes
Source: Diabetologia. 2015 May 30;58(8):1877–86. doi: 10.1007/s00125-015-3627-3 (PMC4499112; doi:10.1007/s00125-015-3627-3)
Supplement: Supplementary file 2 — (PDF 86 kb) [file 125_2015_3627_MOESM2_ESM.pdf]

a. **Bio-ATB-GTP**

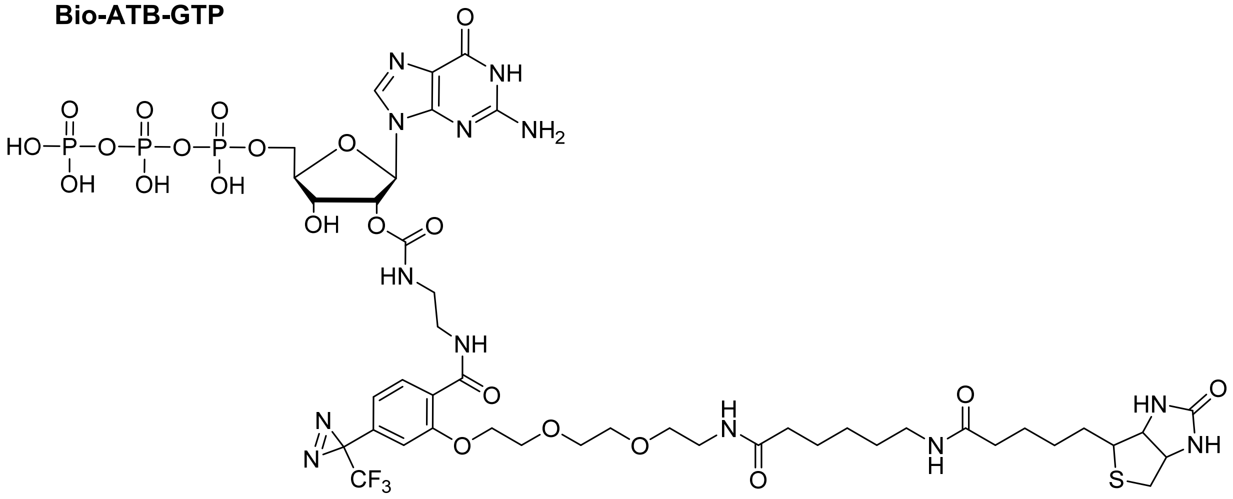

b. **Azidoanilide-GTP biotin-hydrozone**

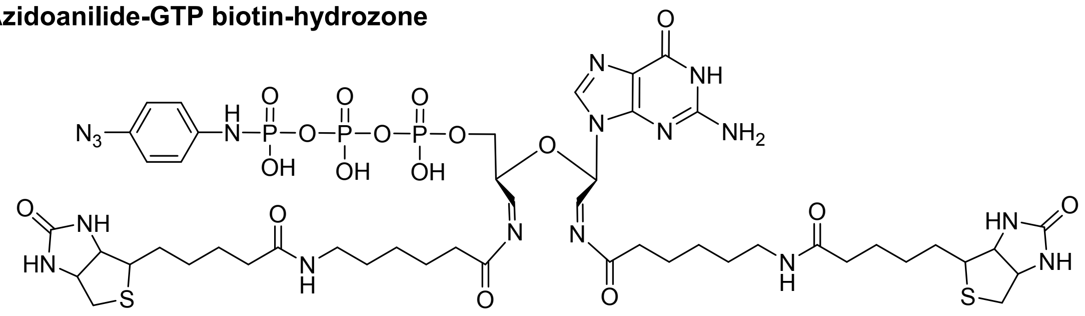

ESM Fig. 1. Structure of GTP photolabels. A. Bio-ATB-GTP is substituted on the ribose hydroxyls 2/3-OH with both the photolabelling diazirine group and a biotin moiety. **B.** Azidoanilide-GTP biotin-hydrozone is substituted in the terminal phosphate of oxidized GTP with a photolabelling azide group while the biotin moiety is substituted onto the ribose aldehydes from oxidation to form biotin hydrazones (the structure is re-drawn from [1]). GTP photolabels that have been used in studies of insulin action include the previously commercially available azidoanilide GTP compound. The biotinylated version of this label has been used to study Rab activation in insulin responsive cardiac [1] and muscle [2] cell lines. These derivatives are substituted with the photoreactive group in the terminal γ-phosphate position of the triphosphate moiety of the GTP. The introduction of the biotin into the azidoanilidyl GTP involves oxidation of the ribose moiety to produce two aldehyde groups which can form adducts to 1, or possibly 2, moles/mol of biotin-aminohexanoate hydrazide ([1] for discussion of the proposed structure). By contrast, we have previously found that the diazirine (azi-trifluoroethyl-benzoate; ATB) photolabels afford distinct advantages over azide photolabels [3]. A diazirine derivative of GTP- with a substitution onto the phosphate terminal γ-S group has been reported [4] while here we report the synthesis and use of a diazirine compound which is substituted with both an ATB and a biotin-amino-hexanoate group into the ribose hydroxyl positions of the GTP. This compound has an un-substituted GTP triphosphate group. This is assumed to allow more sterically unhindered access to the phosphate binding pocket of the studied GTPase. Crystal structures of GppNHp bound Rab3A [5] indicate that the ribose hydroxyls are located on the surface of the structure so that substitution of bulky groups onto the ribose hydroxyls would be expected to be well tolerated. Mg^{2+} is necessary for the correct binding of GTP via the interaction with the

terminal γ-phosphate and the activation of the small GTPases [5,6] and thus will stabilize the binding of Bio-ATB-GTP too.

[1] Schwenk RW, Eckel J (2007) A novel method to monitor insulin-stimulated GTP-loading of Rab11a in cardiomyocytes. Cell Signal 19: 825-830

[2] Sun Y, Bilan PJ, Liu Z, Klip A (2010) Rab8A and Rab13 are activated by insulin and regulate GLUT4 translocation in muscle cells. Proc Natl Acad Sci U S A 107: 19909-19914

[3] Hashimoto M, Hatanaka Y, Yang J, Dhesi J, Holman GD (2001) Synthesis of biotinylated bis(D-glucose) derivatives for glucose transporter photoaffinity labelling. Carbohydr Res 331: 119-127

[4] Kaneda M, Masuda S, Tomohiro T, Hatanaka Y (2007) A simple and efficient photoaffinity method for proteomics of GTP-binding proteins. Chembiochem 8: 595-598

[5] Dumas JJ, Zhu Z, Connolly JL, Lambright DG (1999) Structural basis of activation and GTP hydrolysis in Rab proteins. Structure 7: 413-423

[6] Bourne HR, Sanders DA, McCormick F (1991) The GTPase superfamily: conserved structure and molecular mechanism. Nature 349: 117-127
