# Supplementary material for: Insulin regulates Rab3–Noc2 complex dissociation to promote GLUT4 translocation in rat adipocytes
Source: Diabetologia. 2015 May 30;58(8):1877–86. doi: 10.1007/s00125-015-3627-3 (PMC4499112; doi:10.1007/s00125-015-3627-3)
Supplement: Supplementary file 3 — (PDF 89 kb) [file 125_2015_3627_MOESM3_ESM.pdf]

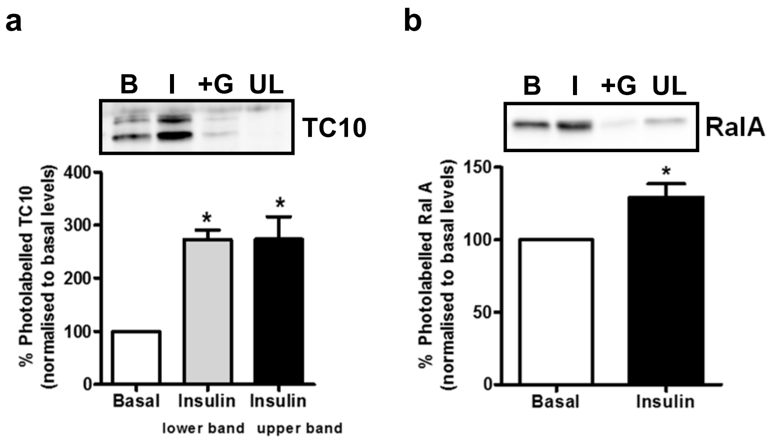

ESM Fig. 2. Photolabelling of GTPases involved in GLUT4 trafficking. Total membrane preparations from rat adipocytes (300 μg/condition) were photolabelled with Bio-ATB-GTP. Biotinylated proteins were streptavidin precipitated and immunoblotted with antibodies against the target proteins: **a.** TC10, **b.** RalA. Data are mean ± SEM from at least 3 independent experiments. * $p < 0.05$ vs basal. The images are representative immunoblots for each set of quantified data. B – basal; I – insulin; +G – 400 fold excess of GTP; UL – no Bio-ATB-GTP added.
