# Supplementary material for: Insulin regulates Rab3–Noc2 complex dissociation to promote GLUT4 translocation in rat adipocytes
Source: Diabetologia. 2015 May 30;58(8):1877–86. doi: 10.1007/s00125-015-3627-3 (PMC4499112; doi:10.1007/s00125-015-3627-3)
Supplement: Supplementary file 4 — (PDF 86 kb) [file 125_2015_3627_MOESM4_ESM.pdf]

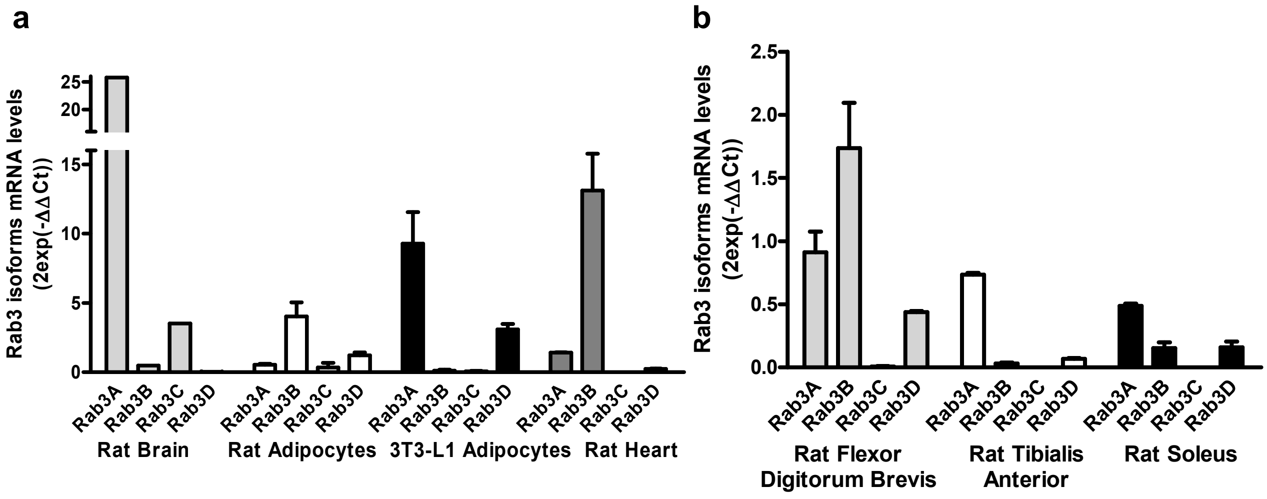

ESM Fig. 3. Rab3 isoforms mRNA levels in insulin sensitive tissues. (a) qRT-PCR analysis of *Rab3 A, B, C* and *D* mRNA in rat brain, rat adipocytes, 3T3-L1 adipocytes and rat heart. Results are mean and SEM from 3 independent experiments. **(b)** qRT-PCR analysis *of Rab3 A, B, C*, and *D* mRNA in *Flexor digitorum breavis*, *Tibialis anterior* and *Soleus* rat skeletal muscle. Data are mean ± SEM from 3 independent experiments.
