# Supplementary material for: Insulin regulates Rab3–Noc2 complex dissociation to promote GLUT4 translocation in rat adipocytes
Source: Diabetologia. 2015 May 30;58(8):1877–86. doi: 10.1007/s00125-015-3627-3 (PMC4499112; doi:10.1007/s00125-015-3627-3)
Supplement: Supplementary file 5 — (PDF 114 kb) [file 125_2015_3627_MOESM5_ESM.pdf]

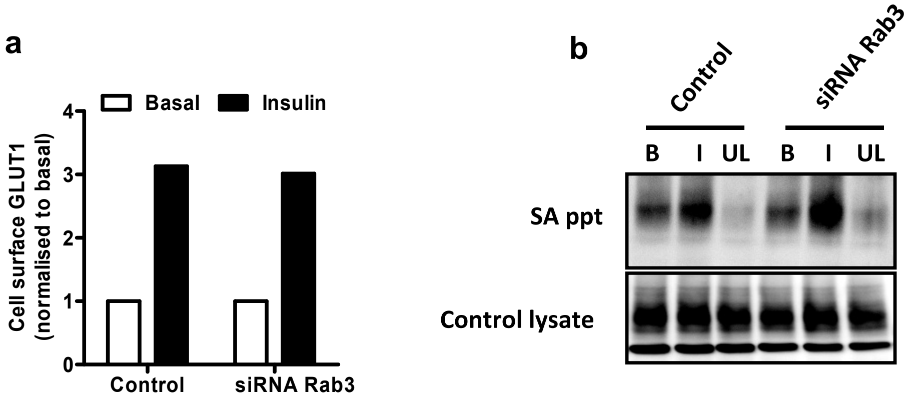

ESM Fig. 4. RNA silencing of all Rab3 isoforms does not affect GLUT1 translocation in 3T3-L1 adipocytes. (**a**) 3T3-L1 adipocytes at day 3 of differentiation were transfected with siRNA against *Rab3A*, *Rab3B* and *Rab3D* (combined). Control cells were transfected with non-targeting siRNA. Untreated or insulin-stimulated cells (100 nM insulin for 30 min) were labelled with Bio-ATB-BGPA. Solubilised and streptavidin precipitated proteins were immunoblotted for GLUT1. Data are mean ± SEM from 2 independent experiments. (**b**) Representative immunoblots for the quantification data in (**a**). SA ppt – streptavidin precipitation, B – basal, I – insulin, UL – no photolabel added.
