# Supplementary material for: Insulin regulates Rab3–Noc2 complex dissociation to promote GLUT4 translocation in rat adipocytes
Source: Diabetologia. 2015 May 30;58(8):1877–86. doi: 10.1007/s00125-015-3627-3 (PMC4499112; doi:10.1007/s00125-015-3627-3)
Supplement: Supplementary file 6 — (PDF 90 kb) [file 125_2015_3627_MOESM6_ESM.pdf]

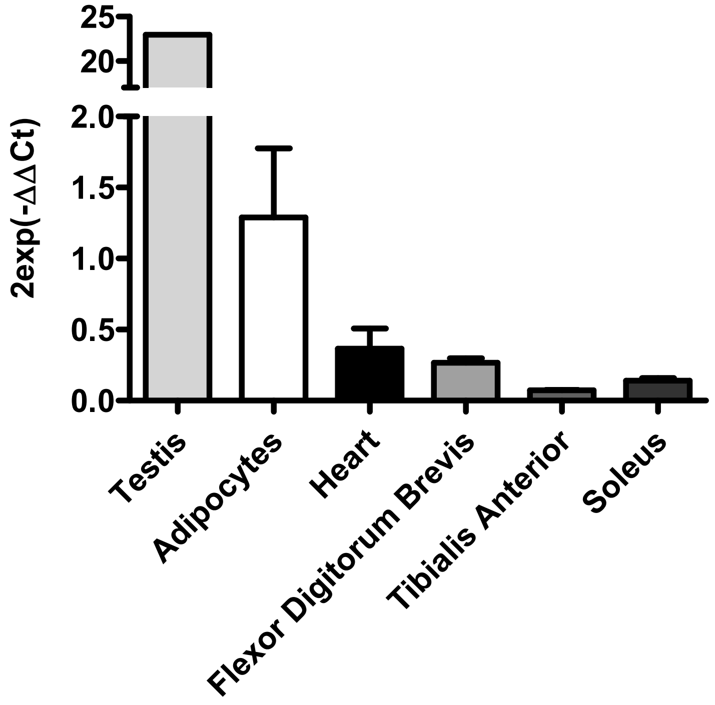

ESM Fig. 5. mRNA levels of *Noc2 (Rph3al)* in rat insulin sensitive tissues. Relative abundance of *Rph3al* mRNA in rat testis, adipocytes, heart, *Flexor digitorum breavis*, *Tibialis anterior* and *Soleus* skeletal muscles assessed by qRT-PCR. Data are mean ± SEM from 3 independent experiments.
