# Supplementary material for: Insulin regulates Rab3–Noc2 complex dissociation to promote GLUT4 translocation in rat adipocytes
Source: Diabetologia. 2015 May 30;58(8):1877–86. doi: 10.1007/s00125-015-3627-3 (PMC4499112; doi:10.1007/s00125-015-3627-3)
Supplement: Supplementary file 7 — (PDF 53 kb) [file 125_2015_3627_MOESM7_ESM.pdf]

ESM Table 1. List of oligonucleotide primers for qRT-PCR.

**Gene name**	**mRNA Accession number**	**Primer 5' > 3'**<br>**Forward primer, Reverse primer**	**Product size**
Rat Rab3A	NM_013018	GCCAGCGTTGTCTCAGTTTA,<br>AATGAGGTTTTGCCCACACT	144
Rat Rab3B	NM_031091	CAGGTTACTGTGTGGGTGAAGA,<br>TGGGAGTTGTATCTTGGTAAATGA	137
Rat Rab3C	NM_133536	AAGGAAGTCTTTGATGTCAGTGG,<br>TGCAAGTAGCAGGTGTTTATAGATG	127
Rat Rab3D	NM_080580	ACCTGTCCTTCTTGCCTCTTC,<br>GAGAGAAAGGGAGCTGAACTTG	132
Rat Rph3al	NM_133591.2	CCCCTGTGGCTGTGTAAGAT,<br>CTTGGGGAGCCCTTTGTAGA	84
Mouse Rab3A	NM_001166399	AATGCAGTGCAGGACTGGTC,<br>ACACCACTCGCTCATCTTCC	106
Mouse Rab3B	NM_023537.5	CGCCATGGGCTTCATCCTA,<br>TGATCTGAGTGGACCAGTCCT	80
Mouse Rab3C	NM_023852.5	CGCTGTACAAGATTGGTCAACT,<br>ACCCGCTCATCTTCCATGTC	99
Mouse Rab3D	NM_031874.4	ACCGACATGACAAGAGGATCA,<br>TAGCTCCGCGATAGTAGGC	93
Mouse TATA box Binding Protein	NM_013684	TGGAAAAGTTGTATTAACAGGTGCT,<br>TCCCTTTAAGATGGGGTAGATGT	82
Rat 28S rRNA	NR_046246.1	TTCACATCGTGGAGGATGATCT,<br>TACACAGTGAAACTTGCCACC	56
