# Supplementary material for: Insulin regulates Rab3–Noc2 complex dissociation to promote GLUT4 translocation in rat adipocytes
Source: Diabetologia. 2015 May 30;58(8):1877–86. doi: 10.1007/s00125-015-3627-3 (PMC4499112; doi:10.1007/s00125-015-3627-3)
Supplement: Supplementary file 8 — (PDF 63 kb) [file 125_2015_3627_MOESM8_ESM.pdf]

ESM Table 2. Mass spectrometry results

	Protein	Protein accession no.	p*I*/Mass (Da)	Peptide Masses (Da)	Amino acid positions and corresponding sequences	Sequence coverage (%)
MALDI-TOF	Ras-related protein Rab3B	Q63941	4.85/24997	1634.81<br>983.33<br>1634.72<br>1671.69	73-85 LQIWDTAGQERYR<br>**137-143 CDMEEER**<br>**137-149 CDMEEERVIPTEK**<br>**187-202 MSDSMDTDPSVLGASK**	19
ESI-Q-TOF	Ras-related protein Rab3B	Q63941	4.85/24997	1099.66<br>1784.89<br>932.46	25-35 LLIIGNSSVGK<br>**152-167 LLAEQLGFDFFEASAK**<br>**179-186 LVDAICDK**	15
	Ras-related protein Rab11B	Q9ET14	5.64/24588	1206.50<br>979.46<br>1079.56<br>1273.60<br>943.47<br>1288.82<br>1159.65<br>1640.97<br>1020.63	5-13 DDEYDYLFK<br>34-41 NEFNLESK<br>42-51 STIGVEFATR<br>62-72 AQIWDTAGQER<br>75-82 AITSAYYR<br>83-95 GAVGALLVYDIAK<br>96-104 HLTYENVER<br>111-125 DHADSNIVIMLVGNK<br>**167-174 NILTEIYR**	41

[a] Peptides highlighted in bold are unique for the respective Rab isoform.
